# Do Not Think Carefully? Re-examining the Effect of Unconscious Thought on Deception Detection

**DOI:** 10.3389/fpsyg.2019.00893

**Published:** 2019-04-26

**Authors:** Song Wu, Hongyu Mei, Jiali Yan

**Affiliations:** College of Psychology and Sociology, Shenzhen University, Shenzhen, China

**Keywords:** unconscious thought, conscious thought, deception detection, verbal cues, non-verbal cues

## Abstract

Several recent studies have examined the effect of unconscious thinking on deception detection with the hypothesis that unconscious thought increases the ability to discriminate between truth and deception, but these studies yielded conflicting results. The present study aimed to re-examine the effect of unconscious thinking and extend it by adopting both verbal and non-verbal/paraverbal stimuli. We hypothesized that unconscious thought leads to a higher accuracy rate than immediate decision and conscious thought when judging non-verbal/paraverbal stimuli, but not when judging verbal stimuli. In Study 1, we compared unconscious thought with immediate decision by using both video and audio stimuli. In Study 2, we compared unconscious thought with conscious thought by using both video and text stimuli. The results showed that when detecting deception vs. truth, (1) unconscious thought was not better than immediate decision on deception detection in both audio and video conditions (Study 1), and (2) unconscious thought was not better than conscious thought in both video and text conditions (Study 2). The Bayes factor of both studies also showed substantial evidence for null hypothesis (H0) relative to alternative hypothesis (H1). The implications and limitations of the present study are discussed.

## Introduction

Studies about deception detection have found that people are not good at differentiating lies from truth: their detection accuracy is slightly above the level of chance (Bond and DePaulo, 2006, 2008; Vrij et al., 2015). Given various pitfalls and disadvantages of conscious thought, researchers have proposed and found that unconscious thought can improve the accuracy of deception detection (Reinhard et al., 2013). However, this superior effect of unconscious thought could not be replicated by another study (Moi and Shanks, 2015). Therefore, the present study aimed to re-examine the effect of unconscious thought on deception detection and further extend previous results by adopting both video and text stimuli.

Psychologists have summarized several reasons for such a low accuracy of deception detection (Reinhard et al., 2013): (1) individuals cannot consciously process complex information in a short time because of limited cognitive resources (Reinhard et al., 2013); (2) the differences that can be recognized between liars and truth tellers are small (DePaulo et al., 2003); (3) individuals possess incorrect beliefs about how to detect liars (Forrest et al., 2004); and (4) conscious detection is susceptible to cognitive and judgmental biases, such as demeanor bias (Reinhard and Sporer, 2010; Levine et al., 2011). As all these reasons are related to consciousness, it is possible that we could improve the ability to detect deception by restricting conscious thought or promoting unconscious thought.

The unconscious thought theory (UTT) proposed that individuals who engage in unconscious thought, rather than conscious thought, can obtain better results when making complex multi-attribute decisions (Dijksterhuis and Nordgren, 2006). According to UTT, conscious thought refers to target-relevant thought processes that occur when conscious attention is focused on the target task, whereas unconscious thought refers to target-relevant thought processes that occur when conscious attention is focused on other distracting tasks (Dijksterhuis and Nordgren, 2006). In the studies exploring UTT, unconscious thought was manipulated by asking participants to complete a distraction task before making a decision or choice about the target task, and it was found that individuals’ performances on the target task became better after a distraction task (Dijksterhuis et al., 2006; Strick et al., 2010, 2011). Specifically regarding deception detection, Reinhard et al. (2013) conducted five experiments to examine the effect of unconscious thought and found that participants in the unconscious thought condition were better at detecting deception vs. truth than participants in the immediate decision condition (standard condition) and conscious-thought condition.

However, because these results are difficult to replicate, many psychologists have criticized UTT. An initial meta-analysis of 17 studies found no substantial evidence for the unconscious thought effect (Acker, 2008). Subsequently, other meta-analyses on various topics also found no reliable support for the claim that unconscious thought is better than conscious thought (Nieuwenstein et al., 2015; Vadillo et al., 2015). Even studies that obtained significant effects of unconscious thought were criticized for their small sample sizes and low power (Nieuwenstein et al., 2015). Considering these unstable results of unconscious thought, Moi and Shanks (2015) tried to replicate the effect of unconscious thought on deception detection found by Reinhard et al. (2013). They conducted two online experiments and found that unconscious thought did not lead to better performance in deception detection than conscious thought and immediate decision conditions. However, since it is difficult to supervise and control participants’ actual behaviors in online experiments, the results might be influenced by other irrelevant factors. Therefore, the first aim of the present study was to replicate the effect of unconscious thought on deception detection by two laboratory-based experiments.

An effective way to gain a better understanding of the unconscious thought effect is to identify the specific cues or information that are used by unconscious thought to make a decision. In their 5th experiment, Reinhard et al. (2013) found that postural position, facial pleasantness, vocal tension, and unfilled pause length were effective cues that helped participants in the unconscious thought condition to make accurate decisions. Since these four cues are non-verbal or paraverbal cues, it is possible that unconscious thought is more sensitive to non-verbal/paraverbal cues than verbal cues. Coincidentally, there is some evidence supporting that conscious thought is more sensitive to verbal cues. For example, high need for cognition, which is a tendency to engage in effortful thinking, led individuals to use more verbal cues, rather than non-verbal cues in the task of deception detection, which in turn led to a higher accuracy rate (Reinhard, 2010). Moreover, individuals with low motivation are more likely to be distracted and rely on unconscious processes. Additionally, it was found that highly motivated individuals, compared to individuals with low motivation, had a lower accuracy rate when judging video-based deceptive/truthful stimuli (Forrest and Feldman, 2000; Porter et al., 2007), but had a higher accuracy rate when judging text-based deceptive/truthful stimuli (Wu et al., 2015). Based on these results, it is possible that unconscious thought leads to higher accuracy only when processing non-verbal or paraverbal cues, rather than when processing verbal cues. Thus, the second aim of the present study was to examine whether the type of cue influenced the effect of unconscious thought on deception detection.

In summary, we conducted two experiments to replicate and extend previous results on the effects of unconscious thought on deception detection. We hypothesized that unconscious thought leads to a higher accuracy rate than immediate decision and conscious thought when judging non-verbal/paraverbal stimuli, but not when judging verbal stimuli.

## Study 1

In this study, we aimed to replicate previous studies (Reinhard et al., 2013; Moi and Shanks, 2015) by comparing the accuracy of unconscious thought to that of immediate decision on deception detection. We also tried to extend previous results by examining the moderating effect of the medium of the stimuli.

### Method

#### Participants and Design

The participants were 145 students (93 females) aged 17–30 years (*M* = 20.71, *SD* = 1.97). They received 20 China Yuan (CNY) as a reward for participation. CNY is Chinese currency; one yuan was worth approximately $0.164 at the time of the study. The present study employed a 2 (thinking mode: unconscious thought vs. immediate decision) × 2 (medium: video vs. audio) between subjects design. Since Reinhard et al. (2013) found medium to large effect sizes for unconscious thought (e.g., $\eta_{p}^{2}$ = 0.12 in Experiment 1, *d* = 0.56 in Experiment 2, and *d* = 0.96 in Experiment 3), an effect size of *f* = 0.33 was set to determine the sample size. Therefore, the present sample exceeded the minimum size (*n* = 122) recommended by G*Power at 0.95 power (*α* = 0.05).

#### Stimuli

Another 16 participants were invited to produce the stimuli. At the beginning, all participants were asked to complete a questionnaire about travel. Participants selected places where they had previously visited from a sample of 50 cities listed on the questionnaire. All participants selected at least one city. Participants were then told to describe the travel experience to an interviewer. Participants were randomly assigned to as truth tellers or liars. Truth tellers were asked to describe a real experience while traveling to their selected city. Liars were asked to describe a fabricated trip experience in anyone of the cities they did not select. Participants were also told that if the interviewer thought they were telling the truth, they would receive 20 extra CNY as a reward. Otherwise, they would not get this reward.

Subsequently, participants were taken to another room and interviewed by another female experimenter. The interviewer asked all participants the same question: “Please tell me in detail the most impressive travel experience you have had.” A video recorder was set behind the interviewer to record participants’ voices and behaviors during the interview. In fact, all participants received 20 yuan regardless of their performances.

We randomly selected 10 videos from the 16 videos. Of the 10 videos, 5 recounted actual travel experiences and 5 recounted fabricated ones. The video-based stimuli contained both visual and acoustic information, so they included non-verbal, paraverbal, and verbal cues.

To produce the audio-based stimuli, we first transcribed the 10 videos into text and then used software to read the text aloud. Two electronic voices were used and the texts provided by male tellers were read by an electronic male voice, whereas the texts provided by female tellers were read by an electronic female voice. This method enabled the exclusion of paraverbal information and matching duration of video and audio stimuli. While the participants listened to each audio stimulus, a still photo of the target person was presented. This helped participants identify each audio stimulus with a specific person. Therefore, the audio stimuli included only verbal cues.

#### Procedure

Upon arrival, participants were told that they would complete a deception detection task and were randomly assigned to either the immediate decision condition or the unconscious thought condition.

In the immediate decision condition, a standard paradigm of deception detection was adopted. Participants were asked to make an immediate conscious binary judgment—truth or deceit—after being presented with each stimulus. About half of the participants were presented with the video stimuli, and the other participants were presented with the audio stimuli.

In the unconscious thought condition, participants were first asked to watch or listen to all 10 stimuli without making any judgments. Similarly, about half of the participants were presented with the video stimuli, and the other participants were presented with the audio stimuli. After that, participants were asked to complete a 3-min distraction task. The distraction task comprised two standard *Sudoku* puzzles and none of the participants completed both puzzles in 3 min. Then, participants were presented with the 10 tellers’ photos one by one, and were asked to judge whether each stimulus was truthful or deceptive.

After completing all 10 judgments, participants were paid and debriefed by the experimenter.

### Results

The average accuracy of all participants across true and deceptive stimuli was 47.93% [*SD* = 15.13, 95% CI = (45.45%, 50.42%)], which was not significantly different from chance (50%), *t*(144) = 1.65, *p* = 0.102. The accuracies (in percentages) for all conditions are displayed in Table 1.

**Table 1:** Means and standard deviations of accuracy for all conditions in Study 1 and 2.

Study	Condition	Truthful stimuli		Deceptive stimuli		Overall	
		*M*	*SD*	*M*	*SD*	*M*	*SD*
1	Unconscious-video	56.47%	19.37	39.41%	24.86	47.94%	17.37
	Unconscious-audio	61.18%	20.27	35.29%	20.92	48.24%	13.59
	Immediate-video	60.00%	21.91	36.67%	22.68	48.33%	13.63
	Immediate-audio	50.73%	23.71	43.90%	18.01	47.32%	16.13
2	Unconscious-video	77.14%	16.90	50.29%	20.79	63.71%	14.57
	Unconscious-text	69.14%	16.34	46.86%	18.11	58.00%	13.02
	Conscious-video	73.71%	18.64	52.00%	19.52	62.86%	12.96
	Conscious-text	72.57%	21.19	53.14%	18.75	62.86%	14.05

The signal detection analysis was used to test our hypothesis (Stanislaw and Todorov, 1999; Reinhard et al., 2013). In the present study, the *hit* was defined as accurate judgments for deceptive stimuli, whereas the *false alarm* was defined as wrong judgments for true stimuli. Therefore, according to signal detection theory, both discrimination ability $d^{\prime }$ and response bias *C* were calculated for further analyses. The hit and false alarm rates were corrected by adding 0.5 to each cell to avoid having z-scores that were −∞ or +∞ (Snodgrass and Corwin, 1988; Hautus, 1995). $d^{\prime }$ is an indicator of the ability to detect deception from truth and greater scores indicate higher ability. *C* is an indicator of the tendency to make truthful or deceptive judgments. A positive *C* value implies a tendency to judge stimuli as truthful (truth bias) and a negative *C* value implies a tendency to judge stimuli as deceptive (deception bias).

A 2 (thinking mode: unconscious thought vs. immediate decision) × 2 (medium: video vs. audio) analysis of variance (ANOVA) was conducted to assess $d^{\prime }$. The main effect of thinking mode on $d^{\prime }$ was not significant, *F*(1,141) = 0.01, *p* = 0.943, $\eta_{p}^{2}$ < 0.001. The main effect of medium on $d^{\prime }$ was not significant, *F*(1,141) = 0.01, *p* = 0.912, $\eta_{p}^{2}$ < 0.001. The interaction effect of thinking mode and medium on $d^{\prime }$ was not significant, *F*(1,141) = 0.12, *p* = 0.726, $\eta_{p}^{2}$ = 0.001. The means and standard deviations of $d^{\prime }$ in all conditions are displayed in Table 2.

**Table 2:** Means and standard deviations of $d^{\prime }$ and *C* for all conditions in Study 1 and 2.

Study	Condition	$d^{\prime }$			*C*			*n*
		*M*	*SD*	95% CI	*M*	*SD*	95% CI	
1	Unconscious-video	−0.12	0.85	[−0.41, 0.18]	0.21	0.34	[0.09, 0.33]	34
	Unconscious-audio	−0.09	0.66	[−0.31, 0.14]	0.31	0.38	[0.18, 0.44]	34
	Immediate-video	−0.08	0.68	[−0.31, 0.15]	0.29	0.43	[0.14, 0.43]	36
	Immediate-audio	−0.14	0.75	[−0.38, 0.10]	0.07	0.32	[−0.03, 0.17]	41
2	Unconscious-video	0.69	0.72	[0.44, 0.94]	0.33	0.32	[0.22, 0.44]	35
	Unconscious-text	0.37	0.63	[0.15, 0.59]	0.26	0.28	[0.17, 0.36]	35
	Conscious-video	0.61	0.63	[0.39, 0.82]	0.27	0.35	[0.15, 0.39]	35
	Conscious-text	0.64	0.72	[0.40, 0.89]	0.24	0.36	[0.12, 0.37]	35

The same ANOVA was used to assess *C*, and results revealed a non-significant main effect of thinking mode, *F*(1,141) = 1.81, *p* = 0.181, $\eta_{p}^{2}$ = 0.013, a non-significant main effect of medium, *F*(1,141) = 1.00, *p* = 0.319, $\eta_{p}^{2}$ = 0.007, and a significant interaction effect of two factors, *F*(1,141) = 6.41, *p* = 0.012, $\eta_{p}^{2}$ = 0.043. Further analyses showed that the *C* value of unconscious thought was not significantly different from immediate decision in the video condition, *t*(68) = 0.44, *p* = 0.438, *d* = 0.19; by contrast, the *C* value of unconscious thought was greater than that of immediate decision in the audio condition, *t*(73) = 2.97, *p* = 0.004, *d* = 0.68 (see Table 2).

According to the suggestions and method proposed by Dienes and McLatchie (2018), we also perform a Bayesian analysis based on the results of Reinhard et al. (2013). Since we did not find a significant interaction effect of thinking mode and medium, we only compared unconscious thinking with immediate decision. The $d^{\prime }$ difference between unconscious thinking and immediate decision group is 0.03, *SE* = 0.28, *t*(143) = 0.12, *p* = 0.905 in Study 1 and the average $d^{\prime }$ difference between unconscious thinking and control group of the first four experiments^1^ was 0.59 in the study by Reinhard et al. (2013). Therefore, according to (Dienes and McLatchie, 2018), we get B_H(0, 0.59)_ = 0.431, which means that our data provided weak evidence for null hypothesis (H0) relative to alternative hypothesis (H1).

In addition, we used the accuracy rates to perform Bayesian analysis. The detecting accuracy difference between unconscious thinking and conscious thinking group is 0.30%, *SE* = 2.53%, *t*(143) = 0.12, *p* = 0.907 in Study 1 and the average accuracy difference between unconscious thinking and control group of all five experiments was 19.84% in the study by Reinhard et al. (2013). Therefore, we get B_H(0, 19.84)_ = 0.127, which means that our data provided substantial evidence for H0 relative to H1.

#### Additional Analysis

The non-significant unconscious thinking effect may be accounted for by the floor effect—if there are no proper cues available in the stimuli, no one can accurately detect deception from truth. In order to examine this possibility, we excluded stimuli whose total accuracy was lower than 50%, which left only three stimuli: two truth tellers (total accuracy: 72.4 and 67.6%) and one liar (total accuracy: 51%). Based on these three stimuli, a 2 (thinking mode: unconscious thought vs. immediate decision) × 2 (medium: video vs. audio) ANOVA was performed. The results showed: (1) no significant differences between the accuracy in unconscious thinking (*M* = 65.20%, *SD* = 22.63%) and immediate decision (*M* = 62.34%, *SD* = 32.15%), *F*(1,141) = 0.23, *p* = 0.635, $\eta_{p}^{2}$ = 0.002; (2) significant differences between the accuracy in video condition (*M* = 70.95%, *SD* = 21.92%) and audio condition (*M* = 56.89%, *SD* = 31.37%), *F*(1,141) = 8.94, *p* = 0.003, $\eta_{p}^{2}$ = 0.060; and (3) a non-significant interaction effect of thinking mode and medium, *F*(1,141) = 3.62, *p* = 0.059, $\eta_{p}^{2}$ = 0.025.

### Discussion

The results of this study partially support our hypotheses. The unconscious thought did not show any advantage in deception detection compared to immediate decision in both audio and video conditions. The present experiment supported the study by Moi and Shanks (2015), but contradicts the study by Reinhard et al. (2013). Considering these conflicting and unstable results, it is still too early to draw conclusions or to apply unconscious thought to deception detection practices. Another flaw of Study 1 is the possible floor effect. Although our additional analysis showed that the floor effect might not interfere with the non-significant unconscious thinking effect, we tried to overcome it in Study 2.

## Study 2

Study 2 aimed to further replicate Study 1 by comparing unconscious thought with conscious thought. Reinhard et al. (2013) found that participants using unconscious thought had a higher accuracy in deception detection than participants using conscious thought, but Moi and Shanks (2015) found that the detection accuracies were not significantly different between these two thought conditions. Although neither study found that conscious thought was superior to unconscious thought, they had the same flaw that placed conscious thought at a disadvantage. Conscious thought relies on having sufficient and accurate information, but participants in these two studies could only rely on memory that was limited, unreliable, and might be distorted (Koriat et al., 2000). Therefore, the low discrimination ability of conscious thought on deception detection may have resulted from insufficient or wrong memory rather than conscious thought itself. To overcome this flaw, the stimuli were presented again for participants in Study 2 while they were engaging in conscious thought. We also examined whether stimulus medium influences the effect of thinking mode on deception detection.

### Method

#### Participants and Design

The participants were 140 students (80 females) aged 18–25 years (*M* = 20.69, *SD* = 1.86). They received 40 CNY as a reward for participation. The present study employed a 2 (thinking mode: unconscious thought vs. conscious thought) × 2 (medium: video vs. text) between-subjects design. As in Study 1, the present sample exceeded the minimum size (*n* = 122) recommended by G*Power at 0.95 power (*α* = .05).

#### Stimuli

In order to avoid floor effect and keep the number of liars equal to truth tellers, we replaced four videos (three liars and one truth teller) that had the lowest accuracy in Study 1. The video stimuli still comprised 10 videos (five truth tellers and five liars) and the text stimuli were made by transcribing the verbal content of these 10 videos into text. When transcribing, we deleted pauses (e.g., “Uh”) and word repetitions to exclude paraverbal cues. Therefore, the text-based stimuli included only verbal cues.

#### Procedure

The participants were randomly assigned to either the unconscious thought or the conscious thought condition. All instructions were provided before presenting stimuli and all participants were told that they would complete a deception detection task.

In the conscious thought condition, participants were told that each stimulus would be presented twice. Specifically, they were asked to understand what happened in the trip in the first presentation, and, in the second, to think hard and carefully about whether the person was telling the truth or lying. Participants were told that they should make a binary judgment (truth or deceit) about each stimulus immediately after the second presentation. Once participants understood these instructions, stimuli were presented one by one. Participants were randomly assigned to either the video or text condition. For the first presentation of text stimuli, five paragraphs were presented one by one as in the unconscious thought condition; whereas for the second presentation, five paragraphs were presented at the same time to assist participants’ conscious thinking. The second presentation of text lasted 2 min and disappeared immediately after the time was up.

For the unconscious thinking condition, participants were told that each stimulus would be presented only once and they would complete a 2-min distraction task after the presentation of each stimulus. Participants were also told that they would judge whether the stimulus was truthful or deceptive immediately after each distraction task. The distraction task comprised several Sudoku puzzles, the number of which was sufficient to fill 10 distraction tasks (e.g., 20 min). Half of the participants were presented with the video stimuli, and the other half were presented with the text stimuli. To control text-reading time, each text was separated into five paragraphs that were presented one by one. Participants could read the next paragraph by pressing the spacebar, but they could not go back to previous paragraphs.

After completing all 10 judgments, participants were paid and debriefed by the experimenter.

### Results

The average accuracy of all participants across true and deceptive stimuli was 61.86% [*SD* = 13.71, 95% CI = (59.57%, 64.15%)], which was significantly higher than chance (50%), *t*(139) = 10.24, *p* < 0.001, *d* = 1.22. The accuracies (in percentages) for all conditions are displayed in Table 1.

As in Study 1, a 2 (thinking mode: unconscious thought vs. conscious thought) × 2 (medium: video vs. text) ANOVA was conducted to assess $d^{\prime }$. Results showed a non-significant main effect of thinking mode, *F*(1,136) = 0.68, *p* = 0.412, $\eta_{p}^{2}$ = 0.005, a non-significant main effect of medium, *F*(1,136) = 1.51, *p* = 0.221, $\eta_{p}^{2}$ = 0.011, and a non-significant interaction effect of these two factors, *F*(1,136) = 2.41, *p* = 0.123, $\eta_{p}^{2}$ = 0.017 (see Table 2).

Similarly, an ANOVA was used to assess *C* and showed a non-significant main effect of thinking mode, *F*(1,136) = 0.49, *p* = 0.487, $\eta_{p}^{2}$ = 0.004, a non-significant main effect of medium, *F*(1,136) = 0.63, *p* = 0.427, $\eta_{p}^{2}$ = 0.005, and a non-significant interaction effect of those two factors, *F*(1,136) = 0.12, *p* = 0.733, $\eta_{p}^{2}$ = 0.001 (see Table 2).

We also performed similar Bayesian analysis as in Study 1. First, for the $d^{\prime }$, the difference between unconscious thinking and conscious thinking group was −0.19, *SE* = 0.29, *t*(138) = −0.65, *p* = 0.516 in Study 2, and the average $d^{\prime }$ difference between unconscious thinking and conscious thinking group of the first four experiments was 0.60 in the study by Reinhard et al. (2013). Therefore, we get B_H(0, 0.60)_ = 0.289, which means that our data provided substantial evidence for the H0 relative to the H1. Second, for the accuracy rate, the difference in Study 2 was −2.00%, *SE* = 2.32%, *t*(138) = −0.86, *p* = 0.390, and the average accuracy difference of all five experiments in the study by Reinhard et al. (2013) was 20.00%. Then, we get B_H(0, 20)_ = 0.063, which means that our data provided strong evidence for the H0 relative to the H1.

### Discussion

Our hypotheses were partially supported by the results. As with the results of Study 1, we did not find any advantage of unconscious thought on deception detection in both the video and text conditions. The present study overcame a flaw in the study of Reinhard et al. (2013) who found that unconscious thought was better than conscious thought. In their studies, conscious thought was assigned after presenting all truthful/deceptive stimuli and the stimuli were not presented again. In such a situation, participants’ explicit memories about one particular stimulus might be distorted unintentionally or confused with other stimuli. Thus, the efficiency of conscious thought might have been impaired because of the lack of accurate memories.

The total accuracy of the unconscious thinking group was higher in Study 2 than that in Study 1. The most likely reason of this result may be that we used four new stimuli, and these new stimuli could be much easier than the old ones. An alternative explanation is that Study 2 allowed more time—20 min in total—to perform unconscious thinking than Study 1 (3 min total). However, since the main aim of Study 2 was to replicate the unconscious thinking effect when excluding interference of floor effect, it is impossible to conclude which explanation is more plausible. Of course, this question is important for understanding the unconscious thinking effect in deception detection and future studies should address it.

## General Discussion

Across two studies, unconscious thought was found to be no better than immediate decision and conscious thought in all video, audio, and text conditions. In addition to the study by Moi and Shanks (2015), we used different stimuli, different media of the stimuli, participants from a different culture, and a laboratory design to replicate the results of Reinhard et al. (2013), but were not successful. We used a similar method to replicate the two previous studies. Participants were first presented with the stimuli, then they completed the distraction task, and finally they made judgments. However, several methodological differences may explain the inconsistent results.

First, in the two previous studies, participants in the unconscious condition were given the instructions about deception judgment after being presented with the stimuli, but in the present study, they were told before watching stimuli. If participants had made a conscious judgment before the distraction task, it is possible that the unconscious thinking effect was interfered with by the strong conscious thinking and then disappeared. However, the UTT did not propose that individuals must not perform conscious thinking before unconscious thinking (Dijksterhuis and Strick, 2016), and many studies about decision making have found the unconscious thinking effect even when stating the real goal of the task at the beginning of experiments (Dijksterhuis et al., 2009; Abadie et al., 2013). Is deception detection a special domain? This question should be clarified in future studies.

Second, the distraction task is different. A word/non-word searching puzzle was used in the two previous studies, but a mathematic Sudoku puzzle was used in the present study. According to UTT, the less demanding distraction task is more likely to yield the unconscious effect (Dijksterhuis and Strick, 2016). Someone may argue that because Sudoku puzzles are more cognitively demanding than word/non-word searching puzzles, the present study did not find an unconscious thinking effect. In fact, no evidence shows that Sudoku puzzles require more effort than searching puzzles, and we used the simplest Sudoku puzzles that can be completed by college students if there is enough time. In addition, participants were not required or motivated to complete all puzzles. Therefore, we believe that the Sudoku puzzles used in the present study are more likely a less demanding task. Future studies can systematically examine the impact of distraction task difficulty on unconscious thinking effect in deception detection.

Third, the specific stimuli used in the present study are different from the two previous ones. Therefore, one possible reason for these conflicting results is that our stimuli lacked the cues found by Reinhard et al. (2013) to be proper and effective for unconscious thought. However, this reason cannot explain why the advantage of unconscious thought was also not found when Moi and Shanks (2015) directly manipulated these effective cues in their second Experiment. Therefore, at least for now, the effect of unconscious thought on deception detection is unstable and difficult to replicate. Future studies need to focus on the boundaries and requirements of the enhanced effect of unconscious thought on deception detection.

In fact, besides unconscious thought, the unconscious process was also found to increase the ability to detect deception in some other ways. For example, previous studies found that engaging in a distraction task while participants were watching video stimuli increased discrimination ability between truth and deception (Feeley and Young, 2000; Albrechtsen et al., 2009). Moreover, researchers found that unconscious judgment, such as in an implicit association test, was better than traditional conscious binary judgment (ten Brinke et al., 2014; van’t Veer et al., 2015). However, these results were also criticized by other researchers and led to considerable debate (ten Brinke and Carney, 2014; Franz and Luxburg, 2015; Wu et al., 2015; Street and Vadillo, 2016).

To better understand these conflicting results, researchers should construct a more specific theory that focuses on the boundaries and requirements of the unconscious thought effect to explain why and when unconscious processes can increase the ability to detect deception. Initially, researchers should identify which specific cues unconscious and conscious processes use; the present study sheds light on this topic. Although not significant, Study 2 found that the video-unconscious condition obtained the highest discrimination ability and the text-unconscious condition obtained the lowest discrimination ability. This result suggests that non-verbal/paraverbal cues, rather than verbal cues, are more effective for unconscious processes. Previous studies also found that participants engaging in unconscious process were more likely to use non-verbal/paraverbal cues, which in turn led to higher accuracy (Feeley and Young, 2000; Reinhard et al., 2013). By contrast, previous studies found that more deliberate processes caused by higher motivation led to higher detection accuracy if participants focused on and processed verbal rather than non-verbal cues (Reinhard, 2010; Reinhard and Sporer, 2010; Wu et al., 2018). It is suggested that verbal cues, rather than non-verbal/paraverbal cues, are more effective for the conscious process. Therefore, it is possible that there is a cue-process matching effect in which the conscious process is more sensitive to verbal cues and the unconscious process is more sensitive to non-verbal/paraverbal cues. However, since the video stimuli in the present study included verbal, paraverbal, and non-verbal cues, the cue-process matching effect could not be examined directly. Future studies are encouraged to examine this effect directly and find out which specific aspects of verbal cues (or non-verbal/paraverbal cues) are used by the conscious (or unconscious) process.

There are some limitations in the present study. First, the stimuli used in our study were lacking in variety. It is unknown whether conscious thought can also increase the ability to detect other types of lies, such as lying about intentions or lying to help others. Future studies need to adopt various stimuli to examine the effect of conscious/unconscious thought on deception detection. Second, no personality factors were considered in the present study. However, a previous study found that attachment anxiety interacted with motivation in a deception detection task (Wu et al., 2018). This suggested that individuals with different personality traits might perform differently when they use conscious or unconscious processes to detect deception. Future studies could focus on and examine this topic to obtain a better understanding of the unconscious thought effect.

## Ethics Statement

The study design was approved by the IRB of Shenzhen University. All study participants provided informed consent. Authors also confirm that this article adheres to ethical guidelines specified in the APA Code of Conduct as well as the authors’ national ethics guidelines.

## Data Archiving and Sharing

The data can be downloaded *via* https://osf.io/xd4qa/.

## Author Contributions

All authors contributed to the study concept and design. Testing and data collection were performed by HM and JY. SW performed the data analysis and interpretation. SW drafted the manuscript, and HM provided critical revisions. All authors approved the final version of the manuscript for submission.

### Conflict of Interest Statement

The authors declare that the research was conducted in the absence of any commercial or financial relationships that could be construed as a potential conflict of interest.

## Footnotes

^1^In their study, it is impossible to calculate a $d^{\prime }$ from the data of the Experiment 5.
